# Effect of childbirth on the course of Crohn's disease; results from a retrospective cohort study in the Netherlands

**DOI:** 10.1186/1471-230X-11-6

**Published:** 2011-01-26

**Authors:** Marieke Smink, Frederik K Lotgering, Lisette Albers, Dirk J de Jong

**Affiliations:** 1 Department of Obstetrics and Gynaecology, Radboud University Nijmegen Medical Centre, PO box 9101, 6500 HB, Nijmegen, The Netherlands; 2 Department of Gastroenterology and Hepatology, Radboud University Nijmegen Medical Centre, PO box 9101, 6500 HB, Nijmegen, The Netherlands

## Abstract

**Background:**

Pregnant women with Crohn's disease needs proper counselling about the effect of pregnancy and childbirth on their disease. However, Literature about the effect of childbirth on Crohn's disease is limited. This study examined the effect of childbirth on the course of Crohn's disease and especially perianal Crohn's disease.

**Methods:**

This is a retrospective cohort study which was performed in a tertiary level referral hospital in the Netherlands. From the IBD database, female patients aged 18-80 years in 2004 were selected. Data analysis took place in the years 2005 and 2006. Eventually, 114 women with at least one pregnancy after the diagnosis of Crohn's disease were eligible for the study. Differences between groups were analyzed using Wilcoxon Mann Whitney tests and Chi-square analysis with 2 × 2 or 2 × 3 contingency tables. Two-tailed values were used and p values < 0.05 were considered statistically significant.

**Results:**

21/114 women (18%) had active luminal disease prior to pregnancy, with significantly more pregnancy related complications compared to women with inactive luminal disease (Odds ratio 2.8; 95% CI 1.0 - 7.4). Caesarean section rate was relatively high (37/114, 32%), especially in patients with perianal disease prior to pregnancy compared to women without perianal disease (Odds ratio 4.6; 95% CI 1.8 - 11.4). Disease progression after childbirth was more frequent in patients with active luminal disease prior to pregnancy compared to inactive luminal disease (Odds ratio 9.7; 95% CI 2.1 - 44.3). Progression of perianal disease seems less frequent after vaginal delivery compared with caesarean section, in both women with prior perianal disease (18% vs. 31%, NS) and without prior perianal disease (5% vs 14%, NS). There were no more fistula-related complications after childbirth in women with an episiotomy or second degree tear.

**Conclusion:**

A relatively high rate of caesarean sections was observed in women with Crohn's disease, especially in women with perianal disease prior to pregnancy. A protective effect of caesarean section on progression of perianal disease was not observed. However, this must be interpreted carefully due to confounder effect by indication for caesarean section.

## Background

Crohn's disease is a chronic recurrent inflammatory bowel disorder, with a peak incidence between 15 and 35 years of age. Many women with Crohn's disease are in their reproductive years and may choose to become pregnant during their disease. It has been estimated that around 25% of women become pregnant after the initial diagnosis of Crohn's disease [1]. In a subgroup of these pregnant women Crohn's disease is complicated by perianal disease. Women with Crohn's disease need to be counselled about possible maternal and fetal risks during pregnancy and childbirth, as well as the potential consequences for the course of their Crohn's disease in the period after childbirth.

The effects of pregnancy on Crohn's disease and vice versa have been studied and directions for counselling have been formulated [2-7]. The literature, however, is limited about the effects of the mode of childbirth on the course of Crohn's disease especially in those women whose Crohn's disease is complicated by perianal disease [8].

The Royal College of Obstetricians and Gynaecologists ( RCOG ) and The American College of Obstetricians and Gynecologists (ACOG) do not have special guidelines with regard to pregnancy and Crohn's disease. Recently, however, the European Crohn's and Colitis Organisation (ECCO) published guidelines concerning the diagnosis and management of Crohn's disease, including pregnancy [1]. The guideline concerning pregnancy and Crohn's disease recommends vaginal delivery for women with quiescent or mild disease [1]. According to this guideline, an episiotomy should be avoided if possible, because a high rate up to 18% of perianal involvement has been reported [9]. In women with active perianal disease, a caesarean section is recommended by the guideline. However, the advice in the guideline that concerns the mode of childbirth is based on two small studies only [8,9].

To provide the best possible care for the pregnant patient with Crohn's disease, it is important to know what the optimal mode of childbirth is for each individual patient. Caesarean sections are major surgery and consequently are associated with increased hospital costs and maternal morbidity as compared to vaginal delivery [10,11]. Considering the possible harm a caesarean section could do, one may wonder how gastroenterologists and/or obstetricians should counsel their patient with Crohn's disease as to caesarean section for other than obstetric reasons.

Therefore, the primary goal of this study was to examine to what extent the mode of childbirth affects the course of Crohn's disease, especially so in women with perianal disease.

## Methods

### Patients

The department of Gastroenterology of the Radboud University Nijmegen Medical Centre is a tertiary referral centre specialized in Inflammatory Bowel Diseases (IBD). Demographic data from all patients treated at this IBD clinic are prospectively collected in an IBD database. All patients in this database were classified by physician diagnosis according to the Vienna classification [12]. From this IBD database, we selected the cohort of all women between 18 to 80 years of age, known with Crohn's disease in the year 2004. Questionnaires were mailed out in November 2004 to all women in the cohort and chart review with data validation and analysis took place in the years 2005 and 2006. From the responders who fulfilled at least one pregnancy after the diagnosis of Crohn's disease, and who gave their informed consent, the medical records were systematically reviewed for objective details of IBD. For those women in whom childbirth took place in the Radboud University Nijmegen Medical Centre, the obstetric data obtained by questionnaire were verified by obstetric chart review. Included in the analysis of the interrelationship of disease and pregnancy were only the data of the first pregnancy beyond 16 weeks gestational age after the diagnosis of Crohn's disease was made, further mentioned as the index pregnancy. The study was approved by the regional medical review ethics committee.

### Questionnaire

The questionnaire was developed to obtain recall data on IBD and obstetric history. It included detailed questions on the disease, i.e. year of diagnosis, diagnostic tools, disease localization, perianal involvement (fistula and/or abscesses), current medication, surgery, and questions about fertility, number and outcome of pregnancies (miscarriage, ectopic pregnancy or pregnancy beyond 16 weeks gestational age), medication during pregnancy (5-ASA, steroids, immunosuppressive, antibiotics and others (such as infliximab)), pregnancy-related complications ( small for gestational age, pregnancy induced hypertension, intrauterine death or other), mode of childbirth and perinatal outcome. It also included specific questions on the interrelationship of disease and the index pregnancy, i.e. disease activity (active or inactive luminal disease ) prior to and during pregnancy, activity of perianal disease (fistula, abscesses), and if there was progression in the course of their Crohn's disease and/or perianal disease at two years after delivery. Perianal disease was considered active if a woman had producing fistula and/or symptomatic abscesses. In the questionnaire, progression was explained as an increase of gastrointestinal complaints, development of fistula, incontinence, increase in medication, Crohn-related surgery or a combination of these factors. A reminder was sent to the non-responders after three months.

### Statistical analysis

The data from questionnaires and medical records were stored in an Excel database in two-fold and potential discrepancies were compared with the source data. Descriptive analysis of the data was performed with SPSS 12.0. Differences between groups were analyzed using Wilcoxon Mann Whitney tests and Chi-square analysis with 2 × 2 or 2 × 3 contingency tables. Crude Odds ratio's with 95% confidence intervals were calculated for patients with active versus inactive luminal disease prior to the index pregnancy and for patients with versus without peri-anal disease prior to pregnancy. Two-tailed values were used and p values < 0.05 were considered statistically significant.

## Results

### Patients

**Figure 1:**
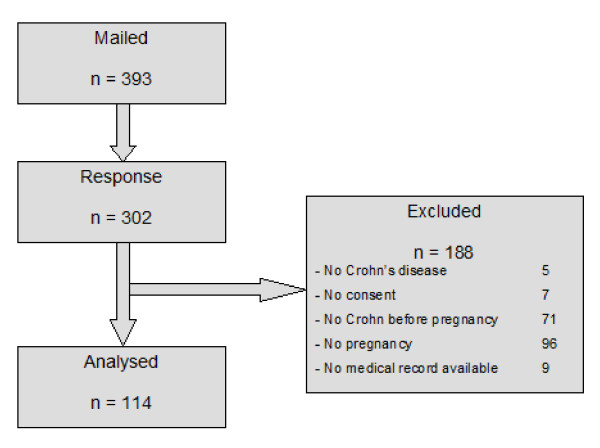
**Flowchart of women included and excluded for analysis**.

Figure 1 depicts the flowchart of women included in the study. From 393 questionnaires that were mailed out to women who satisfied the inclusion criteria, 302 responded. In total, 188 women were excluded from the analysis for the following reasons. At time of the questionnaires, 96 women had no history of childbirth. Furthermore, the diagnose of Crohn's disease was rejected after chart review in 5 patients, no informed consent was obtained in 7 patients, Crohn's disease was diagnosed after pregnancy in 71 patients and in 9 patients, no medical charts were available. Consequently, 114 questionnaires and medical records were available for final analysis. Disease characteristics of these women, obtained from the medical records as well as the data from the questionnaires, are shown in Table 1. The median interval between questionnaire and childbirth is 10 years with a minimum of 1 year and a maximum of 39 years. There were no significant differences in disease characteristics between data obtained from the medical records compared to those from the questionnaires. Information on the 91 non-responders is absent because of lack of consent.

**Table 1:** Disease characteristics of women included, n = 114

	Medical record n (%)	Questionnaire n (%)
Current age Median [range]	44 [34-72]	44 [34-72]

Age of onset Crohn* Median [range]	22 [12-36]	22 [8-37]

Disease location*		
- ileum	27 (24)	29 (25)
- colon	24 (21)	29 (25)
- both	63 (55)	55 (48)
- unknown	0 (0)	1 (1)

Fistula*		
- Yes	63 (55)	52 (45)
- No	51 (45)	43 (38)
- Unknown	0 (0)	19 (17)

Stenosis*		
- Yes	79 (69)	70 (61)
- No	34 (30)	31 (27)
- Unknown	1 (1)	13 (12)

Bowel resection*		
- Yes	78 (68)	80 (70)
- No	36 (32)	33 (29)
- Unknown	0 (0)	1(1)

*No significance between data obtained from medical records and data from questionnaire

### Crohn's disease and no pregnancy

From 96 women without a history of pregnancies while having Crohn's disease in their fertile years, 39 (40%) women were voluntarily childless and 41 (43%) women were involuntarily childless, while in 16 women (17%) the reason for infertility was unknown. Of the women with voluntary childlessness 14 (34%) considered themselves too young to become pregnant at the time of inclusion. More than one third (15/41, 36%) of the women with involuntary childlessness reported severe Crohn's disease as reason of their infertility.

### Crohn's disease and index pregnancy

114 women had been pregnant after the diagnosis of Crohn's disease was made. Of these women, questionnaires and medical records were reviewed. The history revealed that most women (n = 107, 94%) were nulliparous at the time of the index pregnancy, while 7 (6%) women had experienced at least one (vaginal) childbirth. The median duration of Crohn's disease at the birth date of the index child was 8 years (range 0-22). Forty-one (36%) women had one or more miscarriages, and 3 (3%) women reported an ectopic, prior to the index pregnancy. Following the index pregnancy, 56 women had one subsequent pregnancy and 11 had two or more subsequent pregnancies.

#### Pregnancy outcome related to disease activity

The median age at time of delivery in the index pregnancies was 30 years [range 20-40]. Table 2 shows the pregnancy outcome in relation to disease activity prior to pregnancy. Most women (n = 93, 82%) had inactive luminal disease prior to pregnancy and 21 (18%) women had active luminal disease. There were no significant differences in age at index pregnancy or gestational age between the two groups.

**Table 2:** Pregnancy outcome in relation with disease activity prior to pregnancy, n = 114

	Inactive luminal disease 93 (82)	Active luminal disease 21 (18)
Age at index pregnancy Median [range]	30 [20-40]	29 [20-39]

Gestational age in weeks Median [range]	40 [20-42]	39 [27-41]

Pregnancy complications		
- None	70 (75)	11 (52)
- Intrauterine growth retardation	7 (8)	4 (19)
- Pregnancy induced hypertension	2 (2)	1 (5)
- Intrauterine death	3 (3)	0 (0)
- Other	11 (12)	5 (24)

Mode of childbirth		
- Vaginal delivery	64 (69)	13 (62)
○ Spontaneous	47 (73)	9 (69)
○ Instrumental	17 (27)	4 (31)
- Caesarean section:	29 (31)	8 (38)
- Medical advice	19 (66)	2 (25)
- Obstetric reason(s)	10 (34)	4 (50)
- Elective ^†^	0 (0)	2 (25)

Median birth weight in grams* Median [range]	3250 [200-5900]	2910 [980-3970]#

Numbers between brackets are percentages

^**† **^request of patient

*Data on birth weight was missing of 5 women

# p = 0.02

Patients with active luminal disease, as compared with inactive luminal disease prior to pregnancy, had significantly more pregnancy-related complications (Odds ratio 2.8; 95% CI 1.0 - 7.4 and a significantly lower median birth weight (2910 g versus 3250 g, p 0.02). The incidence of caesarean sections did not differ between both groups (Odds ratio 1.4; 95% CI 0.5-3.6).

Sixty-four women (69%) with inactive luminal disease delivered vaginally. Of these women, 8 (12%) had no perineal damage, 42 (66%) an episiotomy, and 14 (22%) a second degree tear. In the group of women with active luminal disease prior to pregnancy 13 (62%) delivered vaginally. Of these women, 1 (8%) had no perineal damage, 10 (77%) an episiotomy and 2 (15%) a second degree tear. There were no third degree tears in either groups.

#### Course of Crohn's disease after childbirth

Table 3 shows the progression of Crohn's disease 2 years after childbirth of women with inactive (n = 93) and active (n = 21) luminal disease prior to pregnancy.

**Table 3:** Long term effect of mode of childbirth on disease activity

	Inactive luminal disease prior to pregnancy n = 93		Active luminal disease prior to pregnancy n = 21	
Mode of childbirth	Vaginal delivery n (%)	Caesarean section n (%)	Vaginal delivery n (%)	Caesarean section n (%)
	64 (69)	29 (31)	13 (62)	8 (38)

Progression Crohn's disease ≤ 2 years post partum:	30 (47)	13 (45)	12 (92)	7 (88)
- Yes	30 (47)	14 (48)	1 (8)	1 (12)
- No	4 (6)	2 (7)	0 (0)	0 (0)
- Not applicable^†^				

† Delivery less than one year ago or data missing

Disease progression within 2 years after childbirth occurred significant more frequently in patients with active luminal disease as compared to inactive luminal disease prior to pregnancy (Odds ratio 9.7; 95% CI 2.1 - 44.3). In women with active luminal disease, the Odds ratio after vaginal delivery was 12.0 (95% CI 1.5 - 98.2) and after caesarean section 7.5 (95% CI 0.8 - 69.9). Disease progression, however, was not significantly different between women who had a vaginal delivery as compared to a caesarean section, both for women with inactive luminal disease (p = 0.87) and active luminal disease (p = 0.72) prior to pregnancy.

In 11 women, a subsequent pregnancy occurred within the follow-up period of 2 years. In 5 (45%) women, no disease progression was reported during follow-up and in 2 (18%) of these women, disease progression was reported before the subsequent pregnancy. In 4 (36%) women disease progression was reported during the subsequent pregnancy.

#### Course of perianal disease after childbirth

Table 4 shows the long term effect of the mode of childbirth on perianal disease. When the study population was divided into women with (n = 27) and without (n = 87) perianal disease, patients with perianal disease prior to pregnancy significantly more frequent had a caesarean section (Odds ratio 4.6; 95% CI 1.8 - 11.4). Progression of perianal disease at 2 years post partum was reported more often in women with prior perianal disease as compared to women without it, respectively 7/27 (26%) and 6/87 (7%) (Odds ratio 4.7; 95% CI 1.4 - 15.6). Remarkably, progression of perianal disease was reported significantly less frequent after vaginal delivery as compared to caesarean section, respectively 5/77 (6%) and 8/37 (22%). The difference remained not significant in the subgroups with and without prior perianal disease, although the sample size was small. Progression was observed in 18% (vaginal) versus 31% (caesarean) and 5% (vaginal) versus 14% (caesarean) in women with and without prior perianal disease.

**Table 4:** Long term effect of mode of childbirth on perianal disease

	Perianal disease prior to pregnancy n = 27		No perianal disease prior to pregnancy n = 87	
Mode of childbirth	Vaginal delivery n (%)	Caesarean section n (%)	Vaginal delivery n (%)	Caesarean section n (%)
	11 (41)	16 (59)	66 (76)	21 (24)

Progression perianal disease ≤ 2 years post partum:	2 (18)	5 (31)	3 (5)	3 (14)
- Yes	9 (82)	9 (56)	58(94)	18 (86)
- No	0 (0)	2 (13)	5 (1)	0 (0)
- Not applicable^†^				

† delivery less than one year ago or data missing

Of the 27 women with perianal disease prior to pregnancy, only 7 (26%) had active perianal disease. Two of them had delivered vaginally and both women reported no progression of perianal disease within 2 years. As for the 5 women who had a caesarean section, 4 reported no progression or regression in perianal disease, and 1 woman reported progression within 2 years after childbirth. Of the 20 women with quiescent perianal disease prior to pregnancy, 11 had a caesarean section and 4/11 (36%) women reported progression in perianal disease within 2 years after the caesarean section. Progression was not observed in any of the 9 women who delivered vaginally.

#### Effect of episiotomy on perianal disease after childbirth

Of the group of 27 women with perianal disease prior to pregnancy, 11 had a vaginal delivery, 9 had an episiotomy and 2 had a second degree tear. Within two years after delivery, 6 women with an episiotomy (6/11, 55%) reported progression of their Crohn's disease, 2 of whom (2/11, 18%) with fistula-related progression. Of the 87 women without perianal disease prior to pregnancy, 66 delivered vaginally, 43 had an episiotomy and 14 had a second degree tear. Progression of their disease was reported in 32/57 women (56%), including 3 fistula (3/57, 5%). From 9 women without perineal damage, 7 reported progression in disease activity without fistulas within 2 years.

## Discussion

In the present study, we observed an overall caesarean section rate of 32% (37/114) in women with Crohn's disease. This is substantially higher than the incidence of caesarean sections in the Netherlands, which amounts to 13,6% [13]. It came to no surprise, however, as a recent meta-analysis showed that women with Crohn's disease are 1,7 times more likely to undergo caesarean section [14]. We observed no difference in the general course of Crohn's disease within two years after childbirth between vaginal deliveries and caesarean sections. This suggests that caesarean section does not prevent worsening of Crohn's disease. Disease progression within 2 years after childbirth was significant more frequent in those patients with active luminal disease compared to women with inactive luminal disease prior to pregnancy and this was independent of mode of childbirth. However, odds ratio's varied widely and because of the small numbers in the subgroups this outcome has to be interpreted carefully. A lower rate of disease progression in quiescent Crohn's disease is in accordance with previous reports examining the predictive value of markers for disease activity [15,16].

Preexistent perianal disease was found in 24% of women. Caesarean section and progression of perianal disease within 2 years after childbirth was more common in these women. If caesarean section would be protective of subsequent disease progression, one would expect a higher rate of progression in the vaginal group. In contrast, progression of perianal disease was observed significantly less frequent after vaginal delivery than after caesarean section, with the same trend in patients with and without perianal disease prior to pregnancy. However, the significant difference in disease progression between both modes of childbirth needs to be interpreted carefully due to a confounding effect by indication for caesarean section. Confounding by indication may occur if the reason for exposure is a risk by itself for the outcome studied [17]. In this case, prior perianal disease may increase the risk for allocation to a caesarean section and also the risk for perianal disease progression by itself.

As for perineal damage due to an episiotomy and/or rupture, all women who delivered vaginally without an episiotomy or second degree tear, had no fistula problems within 2 years after childbirth. In women without prior perianal disease who had perineal damage after childbirth (episiotomy or second degree tear), 3/57 (5%) developed fistula within 2 years of follow-up.

At present, the European Crohn's and Colitis Organization (ECCO) recommend to perform a caesarean section in women with active perianal Crohn's disease [1]. However, this advice is based on small retrospective studies. In general, caesarean section is associated with an increased risk for complications compared to spontaneous vaginal delivery. This may result in a significantly increased risk of postpartum readmission, due to pelvic injury/wounds, obstetric complications, venous disorders and thrombo-embolism and major puerperal infection [10,11]. Therefore, it is relevant to know if caesarean section is truly indicated. Our results are in line with previous findings that also suggest that caesarean section is not protective [8,18]. Rogers et al reported in a small case series that one woman with active perianal disease delivered vaginally without exacerbation of symptoms, while three out of four women experienced recurrent perianal disease after an elective caesarean section [18]. Similarly, IInyckyj et al reported that all five cases with pre-existent perianal disease had worsening of symptoms within one year after delivery, in contrast to only 1 of 39 cases without prior disease who delivered vaginally. Unfortunately the one-year outcomes of 10 caesarean sections was not reported [8].

Another issue in patients with Crohn's disease who will undergo vaginal delivery, is the question if episiotomy may influence perianal disease. Reports on episiotomy in relation to perianal Crohn's disease are scarce. Brandt et al reported that patients with Crohn's disease without pre-existent perineal involvement who delivered vaginally, usually with episiotomy, had 18% chance of developing perianal involvement. However, results were not separated for episiotomy [9], and the high risk of de novo perineal involvement is not supported by our findings. Ilnyckyj et al. reported that one patient (1/11, 9%) with no previous perianal disease developed active perianal disease after episiotomy complicated by third degree tear, whereas none of the 15 patients with prior perianal disease had active perianal disease within one year [8]. A recent Cochrane review [19] has concluded that restrictive episiotomy policies have a number of benefits compared to routine episiotomy. There is less posterior perineal trauma (any injury to posterior vaginal wall, perineal muscle or anal sphincter), less suturing and fewer complications. With restrictive episiotomy, there is no difference in pain and severe vaginal or perineal trauma, but there is an increased risk of anterior perineal trauma (injury to the labia, anterior vagina, urethra or clitoris). The review supports the view that episiotomy is to be recommended for obstetric reasons only.

Population based studies have shown that up to 50% of Crohn's disease patients develop fistulas within a disease course of 20 years. The majority of these fistulas are perianal [20]. The assumption that caesarean section is protective in women with preexistent perianal disease can be doubted. Unfortunately, our data cannot provide the definitive answer if caesarean section is justified in patients with preexistent perianal disease. The retrospective design of our study does not exclude the possibility of confounding by indication for caesarean section and in some cases a long interval between questionnaire and childbirth whereby recall bias can not be ruled out. Despite the high response rate in our study, sample size was small especially in the subgroups. The definitive answer should come from a randomized prospective study comparing results of both modes of childbirth. However, it seems unlikely that such study will ever be performed because of logistic problems of study size and patients noncompliance. Last but not least we have to keep in mind that the incidence of caesarean section in the Netherlands is relatively low compared to other countries and therefore, the results of this study may not be automatically extrapolated to other health care settings.

In the absence of more rigorous data, the results of the present study support the recommendation that in women with inactive luminal disease elective caesarean section should be performed for obstetric indications only, whereas in patients with pre-existent active perineal disease the advantages and disadvantages of caesarean section versus vaginal delivery should be weighted on an individual basis.

## Conclusion

In conclusion, an increased rate of caesarean sections was observed in women with Crohn's disease, especially in those women with perianal disease prior to pregnancy. Disease progression within 2 years after childbirth was significant more frequent in those patients with active luminal disease prior to pregnancy. In women with perianal disease prior to pregnancy, a caesarean section was not protective as to progression of perianal disease. However, this must be interpreted carefully due to confounder effect by indication for caesarean section.

Our results support the recommendation to limit caesarean section for other than obstetric reasons to women with active perianal disease only. In the absence of proof of any protective or deteriorating effect of episiotomy, it would seem good practice to use episiotomy for obstetric indications only.

## Competing interests

The authors declare that they have no competing interests.

## Authors' contributions

MS contributed to the study design, data management and drafted the manuscript. FL participated in the study design and preparation of the manuscript. LA participated in data management and statistical analysis. DJ contributed to study design, review of clinical data and performed the statistical analysis. All authors read and approved the final manuscript.

## Pre-publication history

The pre-publication history for this paper can be accessed here:

http://www.biomedcentral.com/1471-230X/11/6/prepub
